# Molecular mechanisms of biomineralization in marine invertebrates

**DOI:** 10.1242/jeb.206961

**Published:** 2020-05-29

**Authors:** Melody S. Clark

**Affiliations:** British Antarctic Survey, Natural Environment Research Council, High Cross, Madingley Road, Cambridge, CB3 0ET, UK

**Keywords:** Calcification, Calcium carbonate, Climate change, Coral, Mollusc, Urchin

## Abstract

Much recent marine research has been directed towards understanding the effects of anthropogenic-induced environmental change on marine biodiversity, particularly for those animals with heavily calcified exoskeletons, such as corals, molluscs and urchins. This is because life in our oceans is becoming more challenging for these animals with changes in temperature, pH and salinity. In the future, it will be more energetically expensive to make marine skeletons and the increasingly corrosive conditions in seawater are expected to result in the dissolution of these external skeletons. However, initial predictions of wide-scale sensitivity are changing as we understand more about the mechanisms underpinning skeletal production (biomineralization). These studies demonstrate the complexity of calcification pathways and the cellular responses of animals to these altered conditions. Factors including parental conditioning, phenotypic plasticity and epigenetics can significantly impact the production of skeletons and thus future population success. This understanding is paralleled by an increase in our knowledge of the genes and proteins involved in biomineralization, particularly in some phyla, such as urchins, molluscs and corals. This Review will provide a broad overview of our current understanding of the factors affecting skeletal production in marine invertebrates. It will focus on the molecular mechanisms underpinning biomineralization and how knowledge of these processes affects experimental design and our ability to predict responses to climate change. Understanding marine biomineralization has many tangible benefits in our changing world, including improvements in conservation and aquaculture and exploitation of natural calcified structure design using biomimicry approaches that are aimed at producing novel biocomposites.

## Abstract (precis)

**Summary:** Understanding how urchins, molluscs and corals extract calcium from seawater to make their skeletons, and the factors that affect this process, improve predictions on their future success under climate change.

## Introduction

Biomineralization is a fundamental process in the world's oceans; it is the biological mechanism whereby the calcium dissolved in seawater is used to produce solid crystal mineralized structures, namely skeletons and exoskeletons. Biomineralizing animals are ubiquitous to the world's oceans and are incredibly biodiverse, with a wide variety of physiologies, anatomies and habitats. They are vital players contributing to the health and sustainability of marine ecosystems through their contribution to food webs and global biodiversity (Box 1). They are also important in global carbon cycling. For example, our recent geological history has seen the world's oceans become a significant net sink of anthropogenic CO_2_, with the estimation that the world's continental shelves (see Glossary) alone absorb 0.4 Pg carbon year^−1^ (see Glossary) (known as blue carbon) (Bauer et al., 2013). The process whereby our oceans absorb CO_2_ is an important buffer for future increases in atmospheric CO_2_ and therefore an important weapon in global resilience to anthropogenic climate change. Previous estimates of blue carbon have concentrated on calculating the carbon assimilated by microscopic biomineralizers that live in the water column, such as the coccolithophore *Emiliania huxleyi* (Balch et al., 2007). However, there is increasing recognition that the invertebrate macrofauna, such as echinoderms and coralline algae, play significant roles in assimilating and recycling carbon at local and global levels, and are under-represented in the current models (Lebrato et al., 2010; van der Heijden and Kamenos, 2015; Snelgrove et al., 2018). Whilst this role as a carbon sink may be an abstract concept to quantify, certain aspects of biomineralizing species have significant associated tangible economic costs (Box 2), which reinforce the importance for more detailed investigations in these species.

**Glossary**

**Benthic**

The body of water closest to, and including, the sediment.

**Continental shelf**

The area of seabed around a continent that is relatively shallow compared with the open ocean.

**Epibiont**

An organism that lives on the surface of another.

**Epigenetic**

Alteration to the gene expression profile of an organism, which is not reliant upon changes to the DNA. Often called genotype–environment interaction.

**Marine protected areas**

Seas, oceans and estuaries that are protected for conservation purposes.

**Mesenchyme cells**

Pluripotent stem cells, which can give rise to a variety of cell types.

**Metazoa**

Multicellular animals.

**Parental conditioning**

The pre-conditioning of the maternal parent to altered conditions or experimental regimes, which can affect offspring phenotype.

**Pg**

Petagram, equivalent to 10^15^ g or 1 metric gigatonne.

**Phenotypic plasticity**

The physiological flexibility of a species, whereby different phenotypes occur within and between populations in the absence of genetic adaptation; often environmentally induced.

**Physiological tipping point**

The point at which an animal's physiological state suddenly declines.

**Prismatic layer**

Middle shell layer found in some molluscs, which largely consists of calcium carbonate crystals.

**Saturation horizon**

The boundary between shallower waters where calcium carbonate is at saturated concentrations and deeper waters, where calcium carbonate is undersaturated.

**Spicule**

Small, thin pointed element, often containing a high proportion of calcium or silicon. These are structural elements in most sponges.

**Transgenerational plasticity**

Environmentally induced phenotypic plasticity that can be carried over across generations.

**Box 1. The ecological and socio-economic benefits of calcified marine invertebrates**

Whilst there is an increasing realization that marine invertebrates are very effective carbon sinks (as described above), they also contribute to other ecosystem processes. For example, many benthic (see Glossary) biomineralizing species, such as corals and molluscs, are also very effective ecosystem engineers (Bellwood and Hughes, 2001; Gutiérrez et al., 2003). They provide architectural complexity and habitat niches for many marine species. As a result, coral reefs are among the most biodiverse habitats in the world (Bellwood and Hughes, 2001). These benthic species also contribute directly towards measures to mitigate climate change. They can be used to develop artificial reefs that act as natural barriers to combat sea-level rise and increase biodiversity in marine protected areas (see Glossary) (Walles et al., 2016). Calcified marine invertebrates also have socio-economic benefits, such as ensuring water quality, promoting tourism and aquaculture (Box 2). Furthermore, they are a source of bioactive compounds for the biochemical and pharmaceutical industries (Ibañez et al., 2012). Their shell waste is increasingly being used as a renewable calcium source in poultry food and recycled as a component of construction aggregates (Morris et al., 2019). Detailed analyses of the biomineralization processes and the microstructures produced in many species are also providing inspiration for novel materials and biomimetic applications, such as body armour (Green et al., 2015; Yadav et al., 2016). Hence, biomineralizing species also have critical roles to play in the world's economy, both now and in the future (Box 2).

**Box 2. The economic costs of marine invertebrate biomineralizers**

Certain aspects of biomineralizing species have tangible costs associated with them. The three main areas are aquaculture, recreation and biofouling. In 2018, the Food and Agriculture Organization (FAO) of the United Nations reported a world fishery production of 171 million tonnes, a significant proportion of which was due to shellfish (both wild-caught and aquaculturally produced). Figures available for 2016 show that global aquaculture production for molluscs contributed US$ 29.2 billion to the world's economy – however, these are first-catch values, and even more money is generated after downstream processing (FAO, 2018). In terms of revenue made from recreational activities associated with biomineralizing species, a review on the contribution of invertebrates to ecosystem services (Prather et al., 2013) estimated that, in 2010, recreational activities associated with coral reefs generated US$ 11.5 billion annually. These types of activities are clearly positive and beneficial for the world's economy, but they are offset by the costs incurred through biofouling. The blockage of pipes and equipment in the US power industry is estimated to cost around US$60 million per year, and the increased fuel costs for ships due to hull biofouling are suggested to be as high as US$30 billion annually (Jackson, 2008). In addition, biofouling of fish and shellfish cages is a serious problem for aquaculture, accounting for, on average, 14.7% of total operating costs (Adams et al., 2011). Pioneering hull biofoulers include bryozoans, serpulid tube worms and barnacles, and colonization levels can be very high (Jackson, 2008). For example, in instances of hull biofouling in the North Sea, the layer of encrusting species may be up to 30 cm thick, consisting mainly of barnacles (53.6%) and molluscs (27.3%) (Gollasch, 2002). A generally unaccounted cost of ship hull biofouling is the accidental transport of alien invasive species, which colonize new habitats, often to the detriment of the local ecosystem. It has been estimated that 69% of alien invasive species, many of which are biomineralizers, have been transported to their new habitats via ship hulls (Jackson, 2008).

Given both the enduring ecosystem and economic roles of biomineralizing species and the long history of research into these species (Boxes 1 and 2), it is perhaps surprising that they have only recently become more predominant in marine research. This has been largely due to concern, both at a scientific and public level, over ocean acidification (OA) (Box 3). With their heavily calcified exoskeletons and longer life cycles and generation times, marine macrofauna are generally viewed as the species most vulnerable to changing environmental conditions, including OA (Box 3). In contrast, animals such as fish and marine mammals are protected from skeletal dissolution because they have internal skeletons. Moreover, heavily calcified single-celled animals, such as coccolithophores, have rapid generation times, and therefore the potential to adapt much more quickly to changing conditions than multicellular invertebrates (Schlüter et al., 2014).

**Box 3. Ocean acidification**

It is now the 10th anniversary of the publication of the seminal paper ‘Ocean acidification: the other CO_2_ problem’ (Doney et al., 2009). This paper identified a dramatic rise in atmospheric CO_2_ levels since the Industrial Revolution. This has resulted in a decrease in the average pH of the ocean surface from 8.16 to 8.05, and a reduction in the degree of saturation with respect to calcium carbonate (Doney et al., 2009). This acidification process has clear implications for biomineralizing species. Carbonic acid (H_2_CO_3_) is formed when carbon dioxide dissolves in water. This carbonic acid dissociates into bicarbonate (HCO_3_^−^) and a hydrogen ion (H^+^), the latter of which makes the water more acidic. The bicarbonate (HCO_3_^−^) is used as the carbon source by animals to produce calcium carbonate, a process during which another hydrogen ion (H^+^) is produced, which affects the acid–base balance of the cell [see Cyronak et al. (2016a,b) and Waldbusser et al. (2016) for further details]. All these reactions are in equilibrium and the relative concentrations of each product change according to different conditions (pressure, salinity, temperature, pH, etc.). Thus changing conditions can affect the energetic costs of calcification for marine animals, because skeletal production in calcifying animals requires more energy when the saturation state of calcium carbonate minerals in the ocean is reduced. Extracellular ion and acid–base homeostasis are also affected by this process due to the costs of operating calcium channels and proton pumps associated with calcification, which can significantly impact physiological responses (Sanders et al., 2018). Dissolution of skeletons can also occur if the saturation state of calcium carbonate external to the animal becomes undersaturated (Pörtner, 2008). Whilst ocean acidification generated considerable public concern and stimulated much research on calcifying species, it is important to realize that many other factors can influence marine invertebrate skeleton production and maintenance (e.g. Telesca et al., 2018, 2019).

Marine macroinvertebrates are the phyla with the largest number of species in the sea, with molluscs alone comprising 23% of all named marine organisms (Gosling, 2015). These species produce by far the most diverse range of biogenic materials, skeletal structures and microstructures in the ocean, many of which are proving inspirational for biomimetic applications (Box 1). Hence they provide a significant resource for understanding biomineralization processes and a range of tractable model systems for identifying the genetic pathways involved. Whilst research into the effects of OA on marine invertebrates has increased our understanding of biomineralizing processes, we still have relatively little, and very patchy, knowledge on how marine animals take up, sequester and mobilize calcium to produce the wide range of biomineralized skeletons we see in our oceans (Khalifa et al., 2018).

Deeper investigations into the molecular pathways leading to the production of calcified exoskeletons in macroinvertebrates will facilitate a greater understanding into not only how these determine the final composite material, but also what the cellular costs of producing, maintaining and manipulating these skeletons in the sea are. These cellular costs have significant implications for the physiological responses of marine invertebrates to changing environmental conditions, both now and in the future. This Review will synthesize current knowledge on the molecular mechanisms of biomineralization in marine invertebrates with an emphasis on the classes where this process is best described: sea urchins, molluscs and corals. It will also discuss how such fundamental molecular knowledge is critical to understanding how these species will fare in future changing environments.

## The composition of marine invertebrate skeletons

**Fig. 1.**
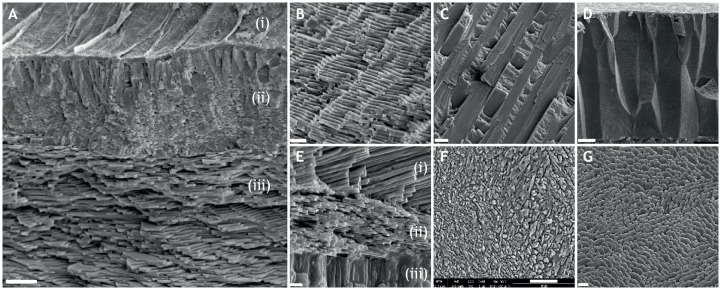
**Different types and arrangements of calcium carbonate microstructures in molluscs.** (A) Scanning electron micrograph of a fracture through the valve of *Myochama anomioides*. Note the organic periostracum on the upper surface (i), with an outer layer of aragonite prisms (ii) and inner layers of sheet nacre (aragonite) (iii). (B) Sheet nacre (aragonite) in *Cleidothaerus albidus*. (C) Crossed-lamellar aragonite in *Ctenioides* *scaber*. (D) Calcite prisms in *Isognomon legumen*. (E) *Mytilus edulis* aragonite nacre (i) and calcite fibrils (ii) and myostracal prisms (iii). (F) Complex crossed-lamellar aragonite in *Mya arenaria*. (G) Foliated calcite in *Ostrea edulis*. Scale bars: A, B and F, 10 µm; C and D, 20 µm; E and G, 2 µm. Photographs courtesy of Elizabeth Harper, University of Cambridge.

The composition of calcified marine macroinvertebrate skeletons, in terms of calcium carbonate crystal structures (polymorphs), crystal arrangements, organic matrix components (proteins), etc. is incredibly varied (Addadi and Weiner, 2014). Individual species do not necessarily contain a single polymorph (Fig. 1). For example, mollusc shells contain different layers consisting of either calcite or aragonite crystals in different microstructures (Carter et al., 2012) and octocorals may have different polymorphs in their skeleton (Bayer and MacIntyre, 2001). Many bryozoans are bimineralic (Taylor et al., 2015). Furthermore, hydrothermal vent species living within the toxic plumes of super-heated water can incorporate additional minerals and metals, such as iron sulphide and silica, into their calcified skeletons, a process which may involve symbiotic chemosynthetic bacteria (Maginn, 2002). These calcium crystals and metal ions are embedded in complex protein matrices, which form an important component of the skeleton, but may also act as calcium carbonate nucleators in addition to constraining crystal growth and determining crystallographic orientation (Marin et al., 2016). The chemical composition, crystal structures and protein content of the calcified skeleton determine not only its physical properties in terms of rigidity and robustness to environmental conditions, but also, critically, the energetic costs of production (e.g. Palmer, 1983, 1992; Meng et al., 2018). If the costs of calcification increase, the energetic budget of the cell may become unbalanced, which can significantly impact the physiology of the animal. A classic example of this is found in the Baltic Sea, where blue mussels in highly saline waters produce much smaller, thinner shells and are less robust as the energetic costs of producing a shell increase strongly with salinity (Sanders et al., 2018).

## The production of marine invertebrate skeletons

The large biodiversity already described in biomineralizing marine invertebrates also extends to the processes by which these external skeletons are made. In some species, specific cells are involved. These include primary mesenchyme cells (see Glossary) in urchins, early mineralizing centres and calicoblast cells in corals and sclerocytes in sponges. In other species with more complex body plans, multiple organs and tissues can be involved. Two prime examples of this are the Crustacea that sequester and mobilize calcium from their chitinous exoskeleton into specialized organs during moulting (Bentov et al., 2016) and molluscs, which produce their remarkable three-dimensional (3D) shell structures via mantle tissue (Kocot et al., 2016; see also ‘The production of 3D structures’ below).

**Fig. 2.**
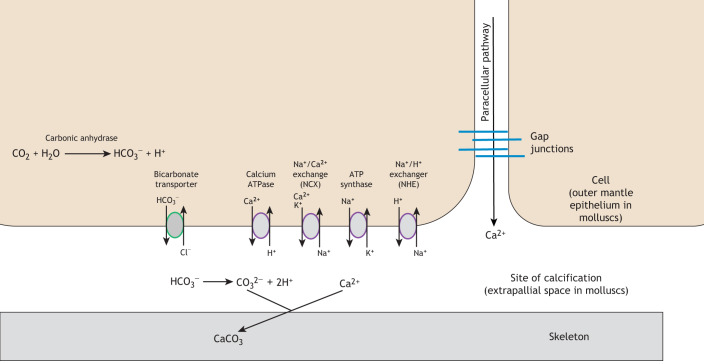
**Schematic representation of the main ion transporters involved in biomineralization at the site of calcification.** Calcium ions entering the calcification site can occur either actively through the transcellular route via transporters (namely calcium ATPase, but also Na^+^/Ca^2+^ exchangers) or less commonly, passively through the intercellular or paracellular pathway. Carbonic anhydrase produces bicarbonate ions within the cell as a by-product of regulating internal pH and water balance. These bicarbonate ions are secreted into the extrapallial space (calcification site) where they react with calcium ions to form calcium carbonate and ultimately the animal skeleton. Other proteins identified are involved in secondary ion transport and maintenance of electrochemical gradients driving physiochemical balance within the cell. Adapted from fig. 2 of Bhattacharya et al. (2016) under Creative Commons Attribution 4.0 International License https://creativecommons.org/licenses/by/4.0/ with additional data from Hofmann et al. (2016), Ramesh et al. (2019) and Sillanpää et al. (2018).

The fabrication of the mollusc shell is almost certainly the result of interactions between biological (genetic and cellular activity, with proteins determining mineral phase, crystal shape and nucleation) and physical processes (crystal competition, growth in confined spaces and self-organization) (Checa, 2018). In bivalve molluscs, a thin layer of mantle tissue encloses all the internal organs with a more complex and substantial outmost edge involved in shell secretion. This mantle edge is divided into a series of folds, with the inner and middle folds mainly involved with water inflow and secretory functions, whilst the outer mantle fold secretes the shell via the outer mantle epithelium (Yonge, 1982). A pivotal structure of this fabrication process is the periostracum. This is a very thin sheet of quinone-tanned proteins, mucopolysaccharides and lipids, secreted by specialized cells between the middle and outer mantle folds (for review, see Harper, 1997). This periostracal sheet reflects dorsally, encompassing the outer mantle fold, defining and enclosing a very narrow space (the extra pallial space), which houses a thin layer of fluid into which the shell is secreted. The periostracum can often be seen on the outside of shells in certain species (e.g. blue mussels and *Myochama anomioides*) (Fig. 1Ai) as a thin dark protein layer, where it also plays a protective role against corrosion. The calcium carbonate shell is predominantly laid down onto the periostracum by the epithelium of the outer mantle epithelium (Harper, 1997). We still have a poor knowledge of how calcium is mobilized within the animal to reach these specialized cells; however, in some species, such as *Crassostrea*, there is evidence to suggest that haemocytes may be involved, but this mechanism is not necessarily universal (Ivanina et al., 2018). Within outer mantle epithelial cells, experiments have demonstrated the active involvement of ion transporters in secreting calcium into the extra pallial space (Fig. 2). For example, in *Crassostrea gigas*, calcium ions passively flow into the outer mantle epithelial cells through voltage-gated calcium channels from the inner mantle side. The calcium is then actively excreted via calcium ATPases and Na^+^/Ca^2+^ exchangers (Fig. 2). The action of the exchangers is driven by the transmembrane sodium gradient created by basal Na^+^/K^+^-ATPases (Sillanpää et al., 2018). Superimposed on this basic cellular mechanism for calcium transport is a regionalization of the mantle tissue and selective secretion of proteins, which results in the plethora of mollusc shell morphologies, as described in ‘The production of 3D structures’ section below.

## The energetic cost of marine invertebrate calcification

It is difficult to calculate the energetic costs of making a calcified exoskeleton because of the problems of separating the costs of accumulating, transporting and precipitating calcium carbonate from routine metabolism. This is due to the overlapping contribution of physiological processes in the maintenance of metabolism, soft tissue growth and calcification (Palmer, 1992). Palmer estimated that the energetic cost of producing a unit of mollusc shell is 1–2 J mg^−1^ of calcium carbonate, but this cost increases dramatically to 29 J mg^−1^ for the production of the proteinaceous organic matrix that often surrounds certain crystal arrangements (Fig. 1) (Palmer, 1983, 1992). These figures have recently been suggested to be on the low side, as they did not really consider the costs of calcium ion mobilization and transmembrane ion transport between cells. These cellular costs may be considerable, particularly in conditions of low salinity, which are associated with a reduction in the availability of calcium and carbonate (Maar et al., 2015; Sanders et al., 2018). However, in spite of these costs, when they are allocated as a proportion of the total energy budget of the animal, they generally account for <5% (Watson et al., 2017; Le Moullac et al., 2018), thus validating observations that it is compensation for exoskeleton dissolution that appears to be the real cost for animals in a changing world (Box 3). But how do we understand these costs and the consequences for the animal in more detail when conditions change? It is here that a knowledge of the molecular pathways underpinning biomineralization can help to identify the different components involved in biomineralization and their costs. Such information can reveal the biochemical pathways that produce specific microstructures and the cellular mechanisms behind responses to a changing environment. Understanding the partitioning of cellular energetics is important in a changing environment, as small shifts in cellular energy budgets can make a significant difference to the physiological state and fitness of the animal.

## Multiple evolutionary events underlie biomineralization in different phyla

Unfortunately, there is no single biomineralizing pathway that is used in the marine environment, which would simplify molecular studies in this area. Independent and convergent evolution of biomineralization has been demonstrated many times at the genomic level in several phyla, including molluscs, corals, brachiopods and sponges (Jackson et al., 2010; Bertucci et al., 2013; Ramos-Silva et al., 2013; Germer et al., 2015; Luo et al., 2015). For example, aragonite biomineralization evolved independently multiple times within a single order of bryozoans, the *Cheilostomata*, whereas nacre building pathways in numerous mollusc lineages are the result of convergent evolution (Jackson et al., 2010; Taylor et al., 2015). Biomineralization has also been achieved via rapid gene evolution in all marine invertebrates studied to date. Transcriptome and proteomic sequencing of skeletal structures and the tissues that produce them has revealed many examples of species- and family-restricted proteins, which often contain unique combinations of ancient co-opted protein domains (reviewed in Kocot et al., 2016). In some species, evolution of biomineralization has also been accompanied by large expansions of gene families. These include chitin-binding proteins, acidic proteins, carbonic anhydrase, repetitive low complexity domain proteins (RLCDs) and tyrosinases (Shen and Jacobs-Lorena, 1999; McDougall et al., 2013; Aguilera et al., 2014; Le Roy et al., 2014; Kocot et al., 2016). Some protein domains, such as carbonic anhydrase, have a long evolutionary history and are present in all calcifying metazoan (see Glossary) species studied to date (Le Roy et al., 2014). Similarly, the chitin-binding domain in invertebrates has a single ancestral origin, but has been duplicated and transposed numerous times within different genes to produce a wide range of variant proteins in different phyla (Shen and Jacobs-Lorena, 1999). In contrast, proteins with specific properties, rather than defined structures, such as acidic-rich proteins and RLCDs (as identified in proteomic studies of marine skeletons) have evolved independently several times within the metazoan lineage (Mass et al., 2013; McDougall et al., 2013). Why these proteins have only evolved in certain species and why some proteins are massively duplicated (and indeed what function each duplicated protein performs) in some species is currently unknown. Many of the genes and proteins listed above have been identified by *in silico* analyses of large sequence datasets; however, many other genes involved in biomineralization in different species have been identified by molecular analyses of animals cultured in different conditions.

## Using experimental manipulation of environmental conditions to uncover biomineralization genes

Over recent years, many laboratory experiments have been carried out on marine invertebrates to identify their responses to environmental change, and OA in particular (Box 3). Initially, the impact of lowering seawater pH or *P*_CO_2__ on skeletal production was largely evaluated by the expression levels of specific genes, namely carbonic anhydrase (e.g. Beniash et al., 2010). The premise was that if the expression level of carbonic anhydrase transcripts was down-regulated under the altered conditions, then calcification must be deleteriously affected. Next-generation sequencing has since dramatically altered our ability to understand the range of cellular responses to changing conditions and identify biomineralization genes. However, the results are complicated to assess because the response of acclimation to the new conditions involves many other biochemical pathways in addition to those involved in biomineralization. Many of the molecular responses of species such as urchins, corals, pteropods (sea butterflies) and molluscs to reductions in seawater pH or *P*_CO_2___ _and/or temperature have revealed significant reductions in gene expression levels associated with a range of functions, namely biomineralization, the cell stress response, cellular energetics and acid–base metabolism (Todgham and Hofmann, 2009; Barshis et al., 2013; Evans et al., 2015; Li et al., 2016; Johnson and Hofmann, 2017; Liu et al., 2017; Maas et al., 2018). Furthermore, when gene expression changes in response to environmental change, it is often difficult to assign genes to biomineralization pathways as many genes are multifunctional (Sleight et al., 2019; Song et al., 2019).

Where the use of experiments to identify biomineralization processes has been particularly useful is in identifying the physiological state of an animal, in terms of health status and homeostasis, and potential energetic trade-offs that affect physiological robustness. For example, in warming experiments with encrusting spirorbid worms it is virtually impossible to determine the physiological state of the animal because of their very small size and their external skeletons. However, molecular analyses in these worms revealed a wholescale lack of acclimation and long-term physiological deterioration of biomineralization and other processes upon chronic temperature increase (Clark et al., 2019a). Developmental time-course experiments in urchins and molluscs have revealed a whole suite of genes involved in biomineralization initiation events, and also the ability of these animals to adopt different calcification strategies for different types of stress (Zhang et al., 2012; Evans et al., 2013). Furthermore, in multiple-stressor experiments, molecular surveys can indicate which stressor has the strongest effect. Experiments in *Crassostrea* species altering either salinity and pH or temperature and pH revealed that pH only produced a relatively minor contribution to changes in gene expression levels under both sets of conditions (Clark et al., 2013; Dickinson et al., 2013). In the case of temperature and pH, animals in warmer conditions were clearly in a poorer physiological condition and were having to mobilize lipid energy reserves to try to maintain homeostasis (Clark et al., 2013). However, these and other experiments in molluscs have indicated that, in multiple-stressor experiments, the additional stress of pH can reduce the tipping point for resilience in a changing world (e.g. Clark et al., 2013; Li et al., 2016).

In all the cases described above, the biomineralization response is identified via the analysis of differentially expressed genes that have been previously implicated in biomineralization pathways, or interrogation for sequence motifs associated with particular gene families in transcriptome data. This means that our knowledge is largely restricted to known, highly conserved sequences with a prior history of involvement in biomineralization as gene annotation is achieved using programs such as Blast2GO (e.g. Johnson and Hofmann, 2017) or bespoke databases of genes and proteins involved in biomineralization (e.g. https://doi.org/10/cz2w) (Yarra, 2018). This has been successful in identifying critical ion transporters in a range of species (Evans et al., 2015; Bhattacharya et al., 2016; Ramesh et al., 2019) and the involvement of known mammalian calcification genes, such as calcitonin G-protein coupled receptors, in invertebrate calcification (Clark et al., 2010; Cardoso et al., 2020). However, to date, the majority of sequencing analyses have concentrated on known biomineralization genes or rather the end-products of biomineralization pathways, and we have little knowledge of the upstream control of these products, or indeed, the interactions between different proteins. This enhanced understanding requires the application of network analyses to transcriptome data. Bioinformatic analysis of gene networks is key to providing a much more holistic understanding of all the genes involved in biomineralization, including those for which there is currently no functional annotation. Although gene regulatory networks have been produced for the purple sea urchin *Strongylocentrotus purpuratus*, particularly during development (e.g. Ettensohn, 2009; Rafiq et al., 2012), such computational techniques are still in their infancy in other species. Recent examples include the application of the correlative weighted gene co-expression network analysis (WGCNA) in the Pacific oyster *Crassostrea gigas*, which highlighted the potential importance of dynein motor proteins as transporters of cellular components during calcification, and an as yet unknown role for protease inhibitors involved in biomineralization (de Wit et al., 2018). The application of ARACHNe (Algorithm for the Reconstruction of Accurate Cellular Networks) to investigate biomineralization in the Antarctic clam revealed novel gene interactions. Carbonic anhydrase interacted with other shell matrix proteins such as Pif and signalling molecules, whereas tyrosinase (another known biomineralization protein) was in a separate gene cluster and was associated with ion transporters (Sleight et al., 2019). The latter study, in particular, highlighted interactions of known biomineralization genes with unannotated sequences, thus incorporating these unknown genes into biomineralization pathways. These more sophisticated types of bioinformatics analyses are key to our future understanding of biomineralization processes and how they affect the wider physiology of the animal. These studies are particularly useful in species where there is already a good understanding of shell mineralogy and microstructure, and a number of marine invertebrate phyla are particularly tractable for investigating different aspects of biomineralization.

## Macroinvertebrates as models for understanding biomineralization

Among calcifying marine species, there are a range of different species (or ‘models’) that have been exploited to enable elucidation of particular aspects of biomineralization. In terms of understanding molecular pathways in any species, the ideal starting resource is a full genome, as this provides the genetic blueprint of the animal. Such genomic data also provide an extensive catalogue of gene and protein sequences that can be used for functional studies, including gene knock-outs, development of antibodies and gene probes for the spatial localization of biomineralization pathway components within cells. However, to date, biomineralizing invertebrates are poorly represented in interactive user-friendly public databases such as those either hosted by Ensembl or using the Ensembl viewer (Table 1). Genome browsers greatly facilitate wider sequence data exploitation, particularly by scientists lacking experience in bioinformatics, who would not necessarily be able to assemble genomes from the raw data deposited in public databases and extract the data they need. Other useful features of biomineralizing model species include tractable cell culture systems, where calcification biochemistry can be studied outside of the complexity of the animal, and the ability to follow individual cell fates. The latter is of prime importance in embryos, where individual cell fates can be linked with the onset of biomineralization. In this respect, urchin embryos have proved incredibly useful.

**Table 1.**
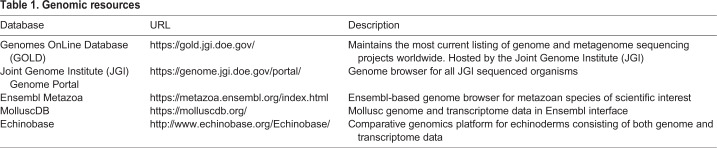
**Genomic resources**

### Developmental models of biomineralization

One of the first marine invertebrates to be sequenced was the purple sea urchin, *S. purpuratus* (Sodergren et al., 2006). Bioinformatic analyses of the genome data revealed distinct differences between the magnesium calcite exoskeleton of *S. purpuratus* and the calcium phosphate skeleton in vertebrates (Sodergren et al., 2006). However, it appears that the molecular processes governing early skeletogenesis events are conserved between echinoderms and vertebrates in terms of the transcription factors and proteins involved in the formation of extracellular matrix (ECM) and cell–ECM interactions (Livingston et al., 2006). Elucidation of these gene interactions has been greatly facilitated by the ease of manipulating *S. purpuratus in vitro* (e.g. Decker et al., 1987)*.* The ability to isolate and follow the development and fates of primary mesenchyme cells into more complex structures, such as spicules (see Glossary), enables the discrete localization and tracing of gene products through development. This has greatly facilitated the identification of biomineralization genes and proteins and their functional characterization in early skeletogenesis (e.g. Killian and Wilt, 2008; Mann et al., 2008, 2010; Killian et al., 2010; Reinardy et al., 2015; Schatzberg et al., 2015). The combination of genomic data with developmental studies has led to the production of extremely sophisticated gene regulatory networks associated with skeletogenesis in this species, which can be used as starting templates for analyses in other species (e.g. Ettensohn, 2009; Rafiq et al., 2012).

### The production of 3D structures

One of the particularly interesting phyla for studying biomineralization is the molluscs. With their unlimited array of shell shapes, colours and combinations of microstructures, molluscs provide an enormous resource for biomineralization research and a plethora of tractable models. Molecular analyses in molluscan species have largely relied on analysing the transcriptome in the mantle, the tissue that produces the calcified shell (e.g. Jackson et al., 2006; Clark et al., 2010; Craft et al., 2010). This situation remains true today, even after the publication of the first mollusc genome, from the Pacific oyster *Crassostrea gigas*, in 2012 (Zhang et al., 2012). The increase in molecular knowledge has enabled scientists to start deciphering how the complex 3D structures of shells are produced. When the expression of genes (identified as being involved in biomineralization) is physically mapped to cells in adult mantle tissue via *in situ* hybridization, a highly regional and cell type-specific pattern emerges (e.g. Sleight et al., 2016; Herlitze et al., 2018). Thus, the activity of individual cells appears to strongly dictate what microstructure is produced, and in what form. This modularity is thought to underpin the wide variety of shell shapes and colours seen in molluscs.

A multitude of proteomic and transcriptomic studies have been conducted on molluscs. However, bioinformatics analyses alone cannot confirm the function of a gene. Here again, molluscs provide valuable model systems. Short-term cell cultures have been used to great effect to characterize the crystallization potential of various proteins *in vitro*. For example, studies in the pearl oyster, *Pinctada imbricata fucata*, have demonstrated the crystallization effects of p10 and pfN23 proteins in nacre and chitin in the prismatic layer (see Glossary) (Zhang et al., 2006; Fang et al., 2012; Kintsu et al., 2017). These types of experiments have largely been adopted by the materials science field in the quest to engineer novel biomaterials and crystal surfaces with unique properties outside of the complexity of the whole animal (e.g. Chang and Evans, 2015). In terms of basic research, gene knock-out experiments are key to identifying gene functionality. There have only been sporadic reports of the successful use of RNAi since 2009, largely in the pearl oyster (e.g. Suzuki et al., 2009). However, the recent demonstration of CRISPR-Cas9 technologies to identify that the *Lsdia1* gene determines shell chirality from the one cell stage of development in the fresh water snail *Lymnaea stagnalis* is a big breakthrough (Abe and Kuroda, 2019). This suggests that CRISPR-Cas9 will be used more frequently in the future to interrogate gene function and to decipher the roles of as yet unidentified genes in biomineralization pathways.

### Identification of ion channels and intracellular calcium transport

Corals are the final macroinvertebrate model phylum, and molecular investigations in these species are aided to a great extent by a comprehensive knowledge of coral skeletal microstructure and the ability to culture specialized biomineralizing cells *in vitro* (e.g. Helman et al., 2008). The main building units of the coral exoskeleton are early mineralizing centres (EMCs) and calicoblast epidermal cells. The calicoblast cells secrete the organic matrix, which forms the protein network around the calcium carbonate crystals of the coral exoskeleton and controls the flux of ions, including calcium, into and out of the cell, which is vital for biomineralization (reviewed in Falini et al., 2015). The calicoblast model system has led to a particularly sophisticated understanding of the cellular ion transporter system associated with their biomineralization, which is one of the most comprehensive studied to date in calcified marine invertebrates. Ion transporter genes identified in calicoblast cells include bicarbonate transporters, voltage-gated calcium channels, carbonic anhydrases and plasma membrane Ca^2+^-ATPases and Na^+^/K^+^-ATPases (Barott et al., 2015; Bhattacharya et al., 2016) (Fig. 2). Many of these ion transporters were initially identified by sequence similarity with known genes, but their function in calcification has since been validated by cellular, physiological and pharmacological inhibitor studies in corals, coralline algae and molluscs (Barott et al., 2015; Bhattacharya et al., 2016; Hofmann et al., 2016; Sillanpää et al., 2018; Ramesh et al., 2019). In coralline algae, there is an additional level of complexity as it appears that light and photosynthesis can significantly affect the action of ion transportation and proton pumps (Hofmann et al., 2016). Also, in corals, any molecular or physiological investigation is complicated due to the presence of symbionts and their complex interactions with their host (Rosic et al., 2015). However, a final advantage of corals is that they are clonal animals that can be successfully split and propagated under different conditions. These clones massively reduce the issue of the high inter-individual variability that is found in wild species, producing less noisy data and the ability to identify more subtle responses when environmental conditions are manipulated (Peck et al., 2015a).

Experiments involving the three phyla described above comprise by far the majority of the molecular analyses conducted on calcifying macroinvertebrates. This is not to say that there is a poor knowledge of biomineralization in other phyla, but molecular studies are much less advanced. Thus, the full variety of biomineralization pathways in marine species has still to be fully uncovered. However, deciphering the molecular pathways of biomineralization is merely the first stage in understanding skeletal and cellular homeostasis, particularly when related to a multitude of different factors that underpin sustainability in future oceans.

### Skeletal sensitivity or resilience in a changing world?

Given the current rate of climate change, future biodiversity in our oceans will be very different from that of today. It is difficult to predict which species will thrive and which will fail in the future, not least because both gradual alteration due to climate change and stochastic events associated with ocean weather need to be considered (Bates et al., 2018). Although the latter are episodic and short-lived, they can take organisms over their physiological tipping point (see Glossary). For example, over recent years, highly publicized environmental catastrophes of biomineralizing species have occurred, including mass bleaching events in scleractinian corals and increased mortality of shellfish due to corrosive upwellings in California (Hoegh-Guldberg, 1999; Feely et al., 2008). Many early predictions of survival of biomineralizing species in OA conditions were based on experimental manipulation of adults and larvae and have often been pessimistic (e.g. Kroeker et al., 2010; Ross et al., 2011; Harvey et al., 2013). However, since the early days of OA research, our knowledge of these calcifying phyla has improved considerably, along with improved experimental design and more integrated multidisciplinary approaches. Many calcified species appear to be more resilient to OA than previously envisaged in early experiments and meta-analyses. For example, brachiopods represent some of the most heavily calcified organisms in our oceans, with their skeletons and calcified support structures comprising greater than 90% of their dry mass. Therefore, they would be expected to be highly sensitive to shell dissolution under future climate scenarios. Long-term OA experiments using different species of live brachiopods have demonstrated a remarkable resilience to lowered seawater pH (Cross et al., 2016; 2019; Ye et al., 2018), thus confounding expectations. This resilience included the production of thicker shells under more corrosive conditions (Cross et al., 2019), although potential energetic trade-offs with reproduction, for example, are unknown. Furthermore, long-term historical studies (100 years plus) of brachiopods and blue mussels showed some alteration to shell microstructure and mineralogy, but no shell dissolution with increasing acidification and warming of the oceans (Cross et al., 2018; L. Telesca, L. S. Peck, T. Backeljau, M. F. Heinig and E. M. Harper, unpublished). These studies confirm some of the previous meta-analyses, which suggested that skeletal dissolution is a more complex process than suggested by OA experiments at the time (Hendriks et al., 2010; Andersson and MacKenzie, 2011). They also affirm the need for long-term monitoring studies, with continual and regular collections of samples (often deposited in, and curated by museums, such as the Natural History Museum in the UK), which provide critical decadal-level data (Cross et al., 2018). Clearly, timescale is very important in the level of animal response. Furthermore, recent analyses of skeletal microstructures are indicating the complexities of dissolution effects.

Calcified exoskeletons are composite biomaterials, the composition of which can vary dramatically even between closely related species. In a recent experiment examining the dissolution of different calcium carbonate exoskeletons, the organic content of these skeletons showed the highest positive correlation with dissolution, indicating that the organic (protein) matrix may be the least resilient feature in a skeleton. However, only 50–60% of the variation in dissolution was explained even when organic composition was considered in combination with other factors, such as magnesium/calcite ratios, crystal density and mineralogy and therefore other, as yet unidentified factors are acting to produce skeletal dissolution (Chadwick et al., 2019). Moreover, we still do not understand how many deep sea scleractinian corals and echinoderms can thrive below the aragonite saturation horizon (see Glossary) at 20–30% calcium carbonate undersaturation (Box 3), conditions where dissolution of exoskeletons would be expected (Flögel et al., 2014). Moreover, as an increasing number of species are studied, we find more examples of resilience to change. In particular, there are certain species of coral, Antarctic urchins and coralline algae, which can internally up-regulate pH at the site of calcification when the external pH of seawater drops, thereby maintaining cellular calcification levels (e.g. McCulloch et al., 2012; Collard et al., 2015; Cornwall et al., 2018). Molecular studies have also shown that pH-resilient populations of the coral *Acropora hyacinthus* permanently express a set of around 60 genes at a higher level than more sensitive populations, and it is these genes that confer their increased physiological robustness (Barshis et al., 2013). This is just one example illustrating that there is sufficient genetic variability in the natural environment for selection to act on and produce more robust populations. Hence, there are still many open questions about skeletal fragility in a changing world.

## Additional factors that impact skeletal production and responses to a changing world

As already described above, biomineralization processes are highly varied and complex, as are the responses of calcified invertebrates to changing conditions. However, there are also other important areas that need to be considered in the design of future experiments that aim to decipher the mechanisms underlying biomineralization. These include assessment of local conditions, phenotypic plasticity (see Glossary), epigenetics (see Glossary) and community level effects.

### The importance of understanding the local environment

Shell composition varies across geographic scales because organisms need to adapt to the constraints of their local environments. This applies not only at large geographical scales (across latitudes) as calcium is more difficult to extract from colder waters (Palmer, 1992; Watson et al., 2012), but also in local habitats, where salinity and food supply play a significant role in determining skeletal composition (Telesca et al., 2018, 2019). Indeed, future food supply and its effect on food webs and, ultimately, biomineralization pathways have been highlighted as a major gap in our knowledge (Branch et al., 2013; Clements and Darrow, 2018). Experiments have shown that unlimited food supplies help molluscs to overcome the increased energetic costs associated with changing conditions, and this will certainly be true in other phyla (Sanders et al., 2013; MacKenzie et al., 2014). Increased food consumption will be needed to fuel both higher metabolic rates in warming seas and increased costs of skeletal maintenance, if adaptation requires the increased production of proteinaceaous components (Palmer, 1992; Leung et al., 2019; Telesca et al., 2018, 2019). These increased costs could also result in the reallocation of energy budgets (energetic trade-offs), which may affect future performance, including reproductive output (e.g. Trussell and Nicklin, 2002; Swezey et al., 2017; Jarrold et al., 2019). The findings showing adaptations to local variability confirm the need to preserve and expand long-term marine monitoring sites as these provide essential real-time data of a changing environment (Poloczanska et al., 2016) and enable mitigation measures to be put in place where needed. A prime example of where monitoring has been successful is in the Pacific north-west shellfish industry, where farmers now choose when to spawn their shellfish according to water conditions and thus avoid the periodic corrosive upwellings, which had previously decimated this industry (Barton et al., 2015).

### Phenotypic plasticity and transgenerational plasticity

The ability to reallocate energetic resources while maintaining physiological performance is often called phenotypic plasticity or physiological flexibility. This concept is considered to be the most important factor in future population sustainability, especially for species with long generation times (Somero, 2010; Peck, 2011). This plasticity of response can be passed onto the next generation via parental preconditioning (see Glossary), which has been demonstrated in a wide range of species, including bryozoans, corals and urchins (Putnam and Gates, 2015; Suckling et al., 2015; Wong et al., 2019). At the genetic level, this is due to adults pre-loading their gametes with transcripts and proteins, which should help the offspring acclimate more readily to the new conditions (Clark et al., 2019b). This plasticity of response can be passed on across generations, a property referred to as transgenerational plasticity (TGP) (see Glossary) (Ross et al., 2016). Recent multigenerational experiments are demonstrating the importance of timescale in responses (as observed in historical studies discussed above). Long-term experiments and multigenerational experiments in particular are revealing how subtle some of these trans-generational effects are (Parker et al., 2015, 2017). How these traits are passed on across generations is currently not known, but epigenetic effects (see Glossary) in response to environmental influences are a potentially important mechanism (Strader et al., 2019).

### Epigenetic effects

Genotype–environment interactions can significantly alter gene expression profiles and physiological functions (reviewed in Eirin-Lopez and Putnam, 2019). Examples of epigenetic modifications in marine invertebrates are scarce. However, recent studies demonstrate the influence of epigenetics on development in oysters, resilience to climate change in corals, acclimation to the inter-tidal lifestyle (including increased biomineralization) in limpets and TGP in urchins (Rivière et al., 2017; Clark et al., 2018; Dixon et al., 2018; Strader et al., 2019). Epigenetics may also be involved in environmental memory. For example, a recent study provided evidence to support the hypothesis that stressors (e.g. low pH) do not have to be continuous to affect organismal physiology (Venkataraman et al., 2019). This finding will clearly be important in experimental design and the interpretation of results considering the growing incidence of, and concern about, the increasing number of episodic heatwaves around the globe (e.g. Oliver, 2019).

### Individual and population level genetic variability

There is still much to discover about the mechanisms underpinning TGP, but even the genetic variation underlying individual variation within a population and the changes in gene expression in response to environmental change are still poorly understood in many species (Gleason, 2019). It is essential to determine if different phenotypes within the same species are due to phenotypic plasticity or individual and population level genetic variation (Vendrami et al., 2017). This variation between individuals and populations was clearly demonstrated in OA studies of *Celleporella* species of bryozoan (Pistevos et al., 2011; Swezey et al., 2017) and in selective breeding experiments of Sydney Rock oysters and the purple sea urchin *S. purpuratus* (Parker et al., 2011; Kelly et al., 2013). In corals, acclimatization and adaptation potential has been shown to be strongly influenced by the genetic background. This can invoke a transcriptome response of constitutive front-loading of ‘useful’ transcripts, which underpins resilience in certain populations (Barshis et al., 2013; Seneca and Palumbi, 2015). Identifying such resilient populations is very important for coral restoration projects and also for identifying resilient populations of shellfish for aquaculture.

### Community-level effects

As demonstrated above, understanding and predicting the biomineralization responses of individual species to change is difficult. This complexity of response is multiplied greatly at the community level, as species-specific responses to biotic and abiotic factors vary considerably, especially when considering their interaction with other species (Hillebrand et al., 2010; Peck et al., 2015b). Due to the inherent difficulties of manipulating temperature in marine systems, the vast majority of experiments study a limited number of species in aquaria (e.g. Peck et al., 2009). However, advances in understanding community interactions are particularly being made with encrusting communities. These studies have been aided by the development of heated settlement panels, which enable temperature manipulation in the sea, allowing encrusting species to develop under as natural conditions as possible (e.g. Ashton et al., 2017; Clark et al., 2019a). Experiments using this technology have demonstrated altered species dominance and variety (Ashton et al., 2017). Predator–prey interactions can also alter competitive interactions and the species balance in the ecosystem (Kroeker et al., 2014). For example, in crustose coralline algae, changes in grazing behaviour in response to predators can impact nutritional intake, which in turn affects physiological status (e.g. Rich et al., 2018). Moreover, in molluscs, shell thickness can increase considerably in the presence of predators, but in marine snails, this reduces the space available for gonads and can affect future fecundity (e.g. Trussell and Nicklin, 2002).

### The microbiome

Community responses must also include the microbiome. Host–microbiota interactions can significantly influence health and sensitivity to disease in humans, and studies are now expanding to other species (Theis et al., 2016). This is a relatively new area of study in marine invertebrates, but is of particular importance for corals and their symbionts (Webster and Reusch, 2017). In these species, the ability to shuffle and switch symbionts when conditions change (a process called microbiome-mediated transgenerational acclimation), can be important and has recently been demonstrated in the Caribbean barrel sponge *Xestospongia muta* and the starlet sea anemone *Nematostella vectensis* (Mortzfeld et al., 2016; Lesser et al., 2016). Even more poorly explored to date is the potential for biofilms and epibionts (see Glossary) to protect the animals they colonize from dissolution (Dery et al., 2018).

Thus, there are numerous factors which impact not only biomineralization processes, but also cellular energetics and physiological responses. These factors need to be taken into consideration in the design of experiments investigating future responses to climate change and when assessing the capacity of individuals, populations and communities to respond to our changing oceans. In ecological experiments, as described here, there are inherent tensions between understanding responses to change and the fundamental mechanisms underpinning biomineralization. This is because studying responses to environmental change is largely concerned with the whole animal response, rather than the fine scale detail of cellular calcium mobilization and biochemistry. However, by encompassing ecological studies within a multidisciplinary framework, scientists from a range of biomineralization fields can benefit from access to tractable models from the marine environment.

### Conclusions and future directions

The importance of biomineralizing species to marine ecosystems is beyond doubt. Their wide biodiversity is mirrored, albeit to a lesser extent, by the breadth of biomineralization research (Endo et al., 2018). As highlighted here with a limited number of species, there will almost certainly be a tractable animal model in our oceans to cover any question related to this subject. Over the years, the sophistication of experiments manipulating marine organisms has not only expanded to include parental conditioning and transgenerational plasticity but also become more multidisciplinary, for example by enabling more accurate tracking of calcium at the cellular level (Fitzer et al., 2019). However, there is still scope for expanding the temporal and spatial scales of individual laboratory experiments. In particular, husbandry over longer periods of time is still problematic in pelagic species (Maas et al., 2018) and natural acidified habitats, if chosen carefully, can provide suitable analogues for the more acidic world of the future (González-Delgado and Hernández, 2018). Greater sequencing of marine invertebrate genomes and deposition in user-friendly databases will definitely facilitate research across a wider range of scientific disciplines. Genomic, transcriptomic and proteomic data are the first stages in deciphering biomineralizing pathways, and in understanding their integration with physiological and ecological responses (Gleason, 2019). Molecular tools, which are standard in model species, barely exist for marine organisms and need development. However, progress is being made in certain areas. The possibility of regulating gene expression via RNAi and using genome editing via CRISPR-Cas9 has recently been demonstrated in corals and molluscs (Clevesa et al., 2018; Funabara et al., 2018; Abe and Kuroda, 2019) and the first computationally predicted gene regulatory network for molluscan biomineralization has been produced (Sleight et al., 2019). These tools are essential for demonstrating gene regulation and protein–protein interactions, but also for identifying putative functions for many of the unannotated genes associated with biomineralization (Funabara et al., 2018; Sleight et al., 2019). The future is certainly promising for marine biomineralization research across a whole range of research areas, and molecular tools will be instrumental to its progress.
